# The Reaction of Diethyl Bromomalonate with *p-tert*-Butylthia-calix[4]arene: An Approach to Asymmetrical Derivatives

**DOI:** 10.3390/molecules14051755

**Published:** 2009-05-07

**Authors:** Omran Abdellah Omran

**Affiliations:** Chemistry Department, Sohag University, Sohag, 82524, Egypt; E-mail: omran2002@mailcity.com

**Keywords:** Thiacalix[4]arene, asymmetry, diethyl bromomalonate

## Abstract

New dissymmetric and asymmetric *p-tert*-butylthiacalix[4]arene derivatives were prepared as a result of the reaction of *p-tert*-butylthiacalix[4]arene with diethyl bromomalonate in the presence of different alkali metals (Cs, K and Na) in refluxing acetone for 7 days. The structures of the prepared compounds were investigated by IR, ^1^H-NMR and MALDI-TOF mass spectroscopy.

## Introduction 

It is well proven that the new member of calixarenes [1,2,3,4,5] family, thiacalixarenes [6,7] (in which the methylene bridged groups has been replaced by sulfur atoms) surpass classical calixarenes as new building blocks because of the novel features offered by the presence of the sulfur atoms. Thiacalixarenes have generated increasing interest both in fundamental and applied chemistry. Many researchers are investigating the chemical behavior of thiacalixarenes as well as using them in different applications via different chemical studies. These studies include: i) modification of the thiacalixarene skeleton through derivatization of the upper and/or lower rims, as well as its sulfur bridged atoms and ii) investigation of the complexation properties of different thiacalixarenes with different chemical species. In this paper, the interaction of thiacalixarene **1** with diethyl bromomalonate in the presence of different alkali metal carbonates (Cs, K and Na) is reported. The functionalization of *p-tert*-butylthiacalixarene **1**, with tetracarbonyl fragments and the remarkable binding ability towards different metals has been investigated [8,9,10]. The idea of this work was to see if we can increase the number of coordinating carbonyl groups by reacting *p-tert*-butylthiacalixarene **1** with diethyl bromomalonate. This might increase the binding ability of the new derivatives.

## Results and Discussion 

**Scheme 1:**
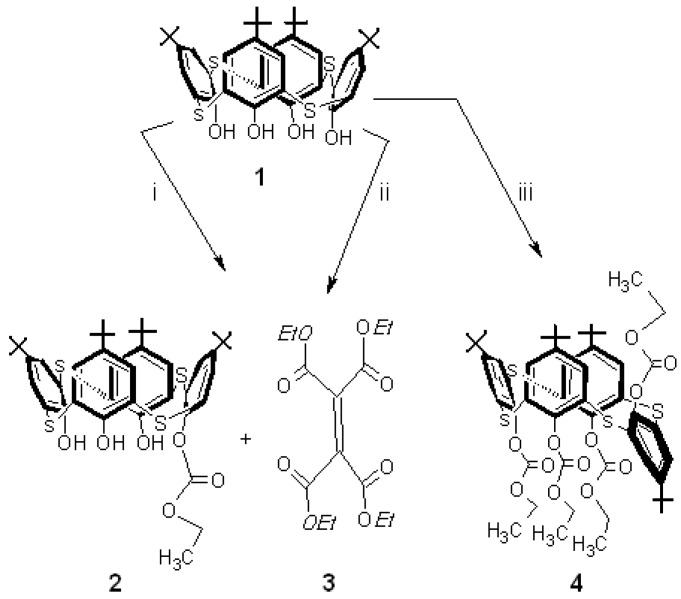
Synthesis of monosubstituted *p-tert*-thiacalix[4]arene **2** (60-70% yield) and tetrasubstituted *p-tert*-butylthiacalix[4]arene **4** (80 % yield).

Previous reported procedures [8,9,10], were used for the first reaction, where *p-tert*-butylthia-calixarene **1** was reacted with diethyl bromomalonate in refluxing acetone in the presence of K_2_CO_3_ as catalyst. Surprisingly, an unprecedented selective synthesis of monosubstituted *p-tert*-butylthiacalixarene **2** containing one ethoxycarbonyl fragment (in 60 % yield) and the reported compound **3** [11] resulted (Scheme 1). When the reaction was carried out using Cs_2_CO_3_ as a base catalyst instead of K_2_CO_3_, a similar result was obtained, with a slight change in the yield of **2** (which increased to 70%). Different spectroscopic analysis (IR, 600 MHz ^1^H-NMR, and MALDI-TOF MS) were used to identify the structure of *p-tert*-butylthiacalixarene **2.** The ^1^H-NMR spectrum exhibits three Bu^t^ group singlets with a relative ratio of 1:1:2, two doublets and two singlets in the aromatic region, one triplet for the CH_3_ group, one quartet for the OCH_2_ group and two singlets of OH group with a relative ratio 2:1, respectively. MALDI-TOF MS shows an m/z peak at 832 (M+K). IR shows bands at 1758 and 3380 cm^-1^ for the CO and OH groups, respectively.

**Scheme 2:**
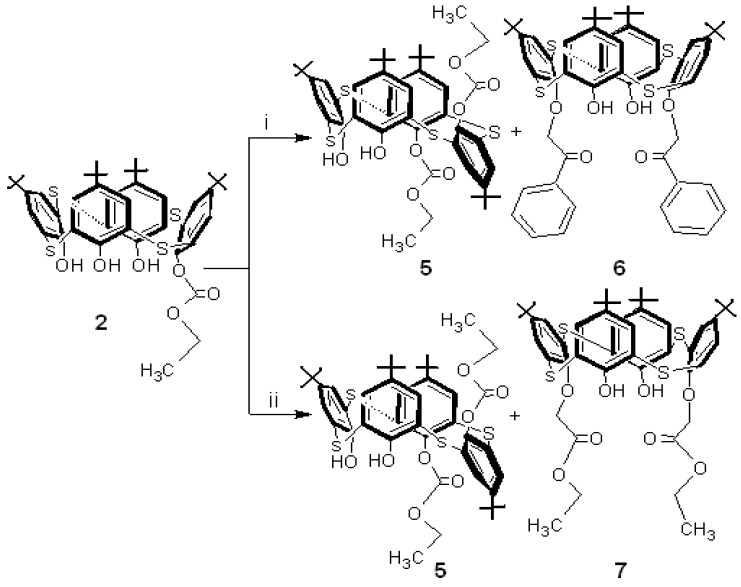
Synthesis of dissymmetric thiacalix[4]arene **5** (40 % yield).

Since the ethoxycarbonyl fragment in *p-tert*-butylthiacalixarene **2** could be introduced to *p-tert*-butylthiacalixarene **1**, through the reaction with ethyl chloroformate, and to investigate the selectivity of the monosubstituted *p-tert*-butylthiacalixarene **2**, ethyl chloroformate was reacted with *p-tert*-butylthiacalixarene **1** in the presence of K_2_CO_3_ in acetone by using the same reaction conditions employed with diethyl bromomalonate. The tetrasubstituted *p-tert*-butylthiacalixarene **4**, was obtained in 80 % yield (Scheme 1; separated by *partial cone* conformer) which is in agreement with the literature [8,9,10]. The structure of **4** was characterized using IR, ^1^H-NMR and MALDI-TOF-MS spectroscopy. The above selective synthesis of the monosubstitued thiacalixarene **2** may be explained by the generation of the ethoxycarbonyl fragment throughout the decomposition of the diethyl bromomalonate in the basic medium or by the attack of the phenolate-metal ion pairs at one of the carbonyl groups of the diethyl bromomalonate and metal ion template effect (Cs and K). This implies that the selective monosubstitution can be attributed to the reaction of thiacalixarene **1** with diethyl bromomalonate. For the preparation of asymmetrical *p-tert*-butylthiacalixarenes, introducing four different groups at the lower rims, monosubstituted thiacalixarene **2** was used for the second step in this study – the reaction of **2** with phenacyl bromide in the presence of Na_2_CO_3_ in acetone (Scheme 2). 

The reaction gave *anti*-1,2-disubstituted *p-tert*-butylthiacalixarene **5** [12] in 40% yield (dissymmetrical thiacalixarene has effective C_2_ symmetry) and the reported disubstituted *p-tert*-butylthiacalixarene **6** [13]. 

**Figure 1:**
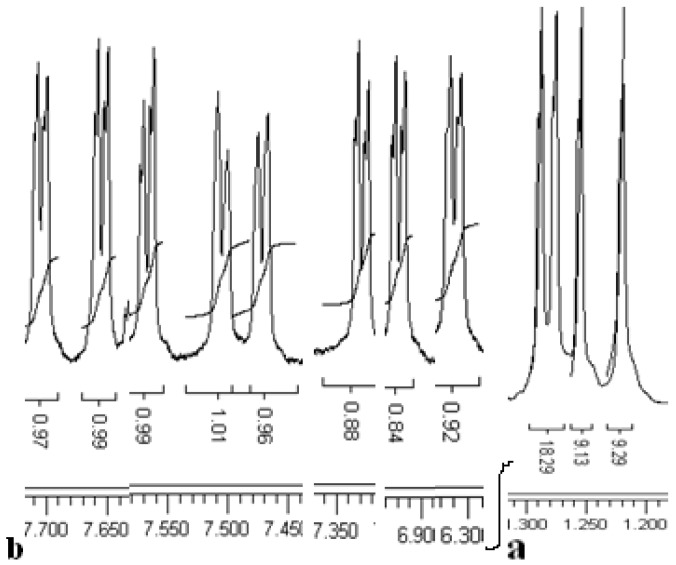
Sections of the ^1^H NMR spectrum of thiacalix[4]arene **5** of Bu^t^ (a) and ArH protons (b).

The ^1^H-NMR spectrum of compound **5** (Figure 1) shows asymmetrical signals for all protons which have been used for the structure determination: four Bu^t^ group singlets, eight doublets with characteristic aromatic proton coupling constants (*J* ~ 2.3 to 2.4 Hz), two OH group singlets, two CH_3_ group triplets, a quartet for the CH_2_, shifted upfield by the aromatic rings and one CH_2_ group multiplet in between two different groups (OH and Ar groups). To confirm the production of *p-tert*-butylthiacalixarene **5**, *p-tert*-butylthiacalixarene **2** was reacted with another alkylation reagent, ethyl bromoacetate and *p-tert*-butylthiacalixarene **5** was formed along with the reported *p-tert*-butyl-thiacalixarene **7** [14] (Scheme 2).

**Scheme 3:**
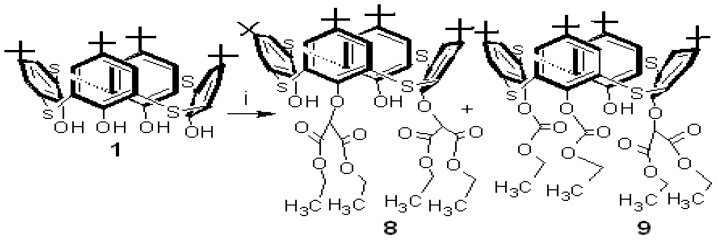
Synthesis of thiacalix[4]arene 8 (30% yield) and asymmetric thiacalix[4]arene 9 (40% yield).

The reaction of *p-tert*-butylthiacalixarene **1** with diethyl bromomalonate in the presence of Na_2_CO_3_ in acetone gave two new *p-tert*-butylthiacalixarenes, **8** and **9**, in 30% and 40% yields, respectively (Scheme 3). 

Both *p-tert*-butylthiacalixarenes **8** and **9** were separated from the carbonate and the acetone layer, respectively. IR, MALDI-TOF-MS and ^1^H-NMR spectroscopic methods were used for determination of the their structures.

**Figure 2:**
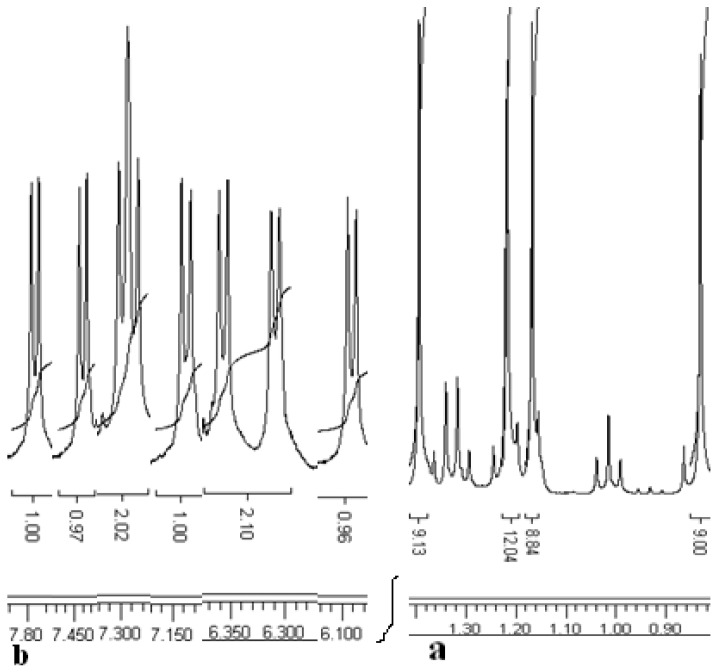
Sections of the Bu^t^ (a) and ArH protons (b)in the^1^H-NMR spectrum of thiacalixarene **9**.

The ^1^H-NMR spectrum of compound **8** exhibits two Bu^t^ group singlets, four multiplets for the methylene groups and four doublets with characteristic meta coupling in the aromatic region (*J* ~ 2.4 Hz). The ^1^H-NMR spectrum of asymmetrical thiacalixarene **9** shows asymmetrical signals for all the types of protons, for instance, four singlets for the Bu^t^ groups, four CH_3_ group triplets, four CH_2_ group multiplets and eight doublets with characteristic coupling constants (*J* ~ 2.3 to 2.8 Hz) in the aromatic region (Figure 2). 

## Experimental

### General

All NMR spectra were recorded on a Bruker DRX600 NMR spectrometer equipped with a triple-gradient TXI (H/C/N) probe operating at a magnetic field strength of 14.1 T., as well as on a Varian Mercury VX-300 NMR spectrometer. All melting points were determined on a Koffler melting point apparatus. IR spectra were obtained on a Nicolet 710 FT-IR spectrometer. Mass spectra were recorded on a MALDI-TOF MS REFLEX III (Bruker-Daltonics, Germany).

### Synthesis of thiacalixarene ***2***

To a suspention of thiacalixarene **1** (2 g, 2.77 mmol) and anhydrous potassium carbonate (6.16 g, 44.32 mmol) and/or cesium carbonate (7.21 g, 22.16 mmol) in dry acetone (100 mL) diethylbromomalonate (8.2 mL, 44.32 mmol) was added. The mixture was refluxed for a week, then it was filtered and separated into two layers: carbonate and acetone. Compound **2** was separated from the carbonate layer after extraction by CH_2_Cl_2_ and obtained as a white solid. Compound **3** was separated from the acetone layer. 

*5,11,17,23-Tetra-tert-butyl-25-[(ethoxycarbonyl)oxy]-26,27,28-trihydroxy-2,8,14,20-tetrathiacalix[4]- arene* (*cone*) (**2**): Yield. 60-70%; Mp: 218 °C; IR (cm^-1^): 1758 (CO), 3380 (OH); MS m/z 832 (M+K); ^1^H-NMR (TMS, 300 MHz, CDCl_3_) δ 0.76 (9H, s, Bu^t^ ), 1.16 (9H, s, Bu^t^), 1.28 (18H, s, Bu^t^), 1.36 (3H, t, *J* =7.1, CH_3_), 4.46 (2H, q, *J* = 7.1, CH_2_), 7.04 (2H, s, ArH), 7.48 (2H, s, ArH), 7.60 (2H, d, *J* = 2.4, ArH), 7.73 (2H, d, *J* = 2.4, ArH), 8.20 (2H, s, OH), 9.10 (1H, s, OH).

### Synthesis of thiacalixarene ***4***

The above method was used for the preparation of **4**, obtained as a white solid. 

*5,11,17,23-Tetra-tert-butyl-25,26,27,28-tetrakis[(ethoxycarbonyl)oxy]-2,8,14,20-tetrathiacalix[4]-arene (partial cone)* (**4**): Yield: 80%; Mp: 330 °C; IR (cm^-1^): 1756 (CO); MS m/z 1072 (MH^+^); ^1^H-NMR (TMS, 300 MHz, CDCl_3_) δ 1.09 (18H, s, Bu^t^ ), 1.38 (9H, s, Bu^t^), 1.47 (9H, s, Bu^t^), 1.33-1.50 (12H, m, CH_3_), 4.19 (2H, q, *J* = 7.0, CH_2_), 4.26-4.48 (6H, m, CH_2_), 7.18 (2H, d, *J* = 2.4, ArH), 7.44 (2H, s, ArH), 7.45 (2H, d, *J* = 2.4, ArH), 7.78 (2H, s, ArH).

### Synthesis of thiacalixarenes ***5**, **6** and **7***

Thiacalixarene **2** (0.6 g, 0.75 mmol) and Na_2_CO_3_ (0.1 g, 0.94 mmol) were suspended in acetone (50 mL) and phenacyl bromide (0.15 g, 0.75 mmol) or ethyl bromoacetate (0.1 mL, 0.9 mmol) were added. The mixture was refluxed for 24 hours. The reaction mixture was concentrated almost to dryness and the solid residue was separated by column chromatography using a 50/50 *n*-hexane-CH_2_Cl_2_ mixture as eluent, to separate the thiacalixarenes **5** and **6** from the reaction with phenacyl bromide; or thiacalixarenes **5** and **7** from the reaction with ethyl bromoacetate.

*5,11,17,23-Tetra-tert-butyl-25,26-bis[(ethoxycarbonyl)oxy]-27,28-dihydroxy-2,8,14,20-tetrathiacalix-[4]arene (anti-1,2-alternate)* (**5**) : Yield: 40%; yellow solid; Mp: 310 °C; IR (cm^-1^): 1754 (CO); MS m/z 889 (M+Na); ^1^H-NMR (TMS, 300 MHz, CDCl_3_) δ 1.07-1.17 (6H, m, CH_3_), 1.22 (9H, s, Bu^t^), 1.26 (9H, s, Bu^t^), 1.28 (9H, s, Bu^t^), 1.29 (9H, s, Bu^t^), 3.7 (2H, q, *J* = 3.7, CH_2_), 4.14-4.34 (2H, m, CH_2_), 6.31 (2H, d, *J* = 2.3, ArH), 6.92 (2H, q, *J* = 2.3, ArH), 7.33 (2H, d, *J* = 2.4, ArH), 7.39 (1H, s, OH), 7.47 (2H, d, *J* = 2.4, ArH), 7.51 (2H, d, *J* = 2.4, ArH), 7.57 (2H, d, *J* = 2.4, ArH), 7.66 (2H, d, *J* = 2.4, ArH), 7.71 (2H, d, *J* = 2.4, ArH), 7.81 (1H, s, OH).

### Synthesis of thiacalixarenes ***8*** and ***9***

The same procedure for the preparation of thiacalixarene **2** was used. Thiacalixarene **8** was separated from the carbonate layer and thiacalixarene **9** was separated from the acetone layer.

*5,11,17,23-Tetra-tert-butyl-25,26-bis[(1,1-diethoxycarbonyl)methoxy]-27,28-dihydroxy-2,8,14,20-tetrathiacalix[4]arene (cone)* (**8**): Yellow solid; Mp: 330 °C; Yield: 30%; IR (cm^-1^): 1751 (CO); MS m/z 1061 (M+Na); ^1^H-NMR (TMS, 300 MHz, CDCl_3_) δ 0.96 (6H, t, *J* = 7.1, CH_3_), 1.18 (6H, t, *J* = 7.1, CH_3_), 1.21 (18H, s, Bu^t^ ), 1.23 (18H, s, Bu^t^), 3.72 (2H, m, CH_2_), 3.79 (2H, m, CH_2_), 3.93-4.04 (6H, m, CH_2_ + OCH), 6.05 (2H, q, *J* = 2.4, ArH), 6.87 (2H, d, *J* = 2.4, ArH), 7.27 (2H, s, OH), 7.44 (2H, d, *, J* = 2.4, ArH), 7.65 (2H, d, *J* = 2.4, ArH).

*5,11,17,23-Tetra-tert-butyl-25-[(1,1-diethoxycarbonyl)methoxy]-26,28-bis[(ethoxycarbonyl)oxy-26-hydroxy-2,8,14,20-tetrathiacalix[4]arene (cone)* (**9**): Yellow solid; Yield: 40%; Mp: 275 °C; IR (cm^-1^): 1758 (CO); MS m/z 1047 (M+Na); ^1^-NMR (TMS, 300 MHz, CDCl_3_) *δ* = 0.84 (9H, s, Bu^t^ ), 1.01 (6H, t, *J* = 7.1, CH_3_) 1.17 (9H, s, Bu^t^) 1.22 (9H, s, Bu^t^), 1.22 (6H, t, *J* = 7.1, CH_3_), 1.33 (12H, m, CH_3_), 1.4 (9H, s, Bu^t^), 3.98- 4.50 (10H, m, CH_2_ + OCH, OH), 6.10 (2H, d, *J* = 2.3, ArH), 6.31 (2H, q, *J* = 2.3, ArH), 6.36 (2H, d, *J* = 2.3, ArH), 7.14 (2H, d, *J* = 2.5, ArH), 7.29 (2H, d, *J* = 2.8, ArH), 7.30 (2H, d, *J* = 2.8, ArH), 7.44 (2H, d, *J* = 2.5, ArH), 7.80 (2H, d, *J* = 2.5, ArH).

## Conclusions 

*p-tert*-Butylthiacalix[4]arenes mono-, di-, tri- and tetra-substituted at the lower rim in different conformers have been prepared as a result of the reaction of *p-tert*-butylthiacalix[4]arene with diethyl bromomalonate in the presence of different alkali metals (Cs, K and Na) in acetone. Two of these derivatives are dissymmetrical and asymmetrical and could have potential use for chiral discrimination.

## Figures and Tables

*Reagents and conditions*: (i) BrCH(COOEt)_2_/K_2_CO_3_, acetone; (ii) BrCH(COOEt)_2_/Cs_2_CO_3_, acetone; (iii) ClCOOEt/K_2_CO_3_, acetone.

*Reagents and conditions*: (i) PhCOCH_2_Br/Na_2_CO_3_, acetone; (ii) BrCH_2_COOEt/Na_2_CO_3_, acetone.

*Reagent and conditions*: (i) BrCH(COOEt)_2_/Na_2_CO_3_, acetone.

## Supplementary Files

Supplementary File 1
