# Omran, O.-A. The Reaction of Diethyl Bromomalonate with *p-tert-*butylthiacalix[4]arene: An Approach to Asymmetrical Derivatives. *Molecules* 2009, *14*, 1755-1761

**DOI:** 10.3390/molecules14114689

**Published:** 2009-11-19

**Authors:** Omran Abdellah Omran

**Affiliations:** 1 Faculty of Science at Zulfi, Qassim University, P.O. Box: 1712, 11932, Saudi Arabia; 2 Chemistry Department, Sohag University, Sohag, 82524, Egypt; E-Mail: omran2002@mailcity.com

We found following errors in our paper published in Molecules recently [1]. On page 1755, the author’s correct affiliation is indicated below:

^1^ Faculty of Science at Zulfi, Qassim University, P.O. Box: 1712, 11932, Saudi Arabia
^2^ Chemistry Department, Sohag University, Sohag, 82524, Egypt; E-Mail: omran2002@mailcity.com
