# Supplementary material for: The Reaction of Diethyl Bromomalonate with p-tert-Butylthia-calix[4]arene: An Approach to Asymmetrical Derivatives
Source: Molecules. 2009 May 7;14(5):1755–61. doi: 10.3390/molecules14051755 (PMC6254204; doi:10.3390/molecules14051755)
Supplement: Supplementary File 1 [file molecules-14-01755-s001.pdf]

Molecules **2009**, *14*, 4689; doi:10.3390/molecules14114689

OPEN ACCESS

molecules

ISSN 1420-3049

www.mdpi.com/journal/molecules

Correction

Omran, O.-A. The Reaction of Diethyl Bromomalonate with *p-tert*-butylthiacalix[4]arene: An Approach to Asymmetrical Derivatives. *Molecules* 2009, *14*, 1755-1761

Omran Abdellah Omran [1,2,*]

[1] Faculty of Science at Zulfi, Qassim University, P.O. Box: 1712, 11932, Saudi Arabia

[2] Chemistry Department, Sohag University, Sohag, 82524, Egypt; E-Mail: omran2002@mailcity.com

* Author to whom correspondence should be addressed; E-Mail: omran2002@mailcity.com; Tel.: +96-6-4226497; Fax: +96-6-4227184.

Received: 17 November 2009/ Published: 19 November 2009

We found following errors in our paper published in Molecules recently [1]. On page 1755, the author's correct affiliation is indicated below:

[1] Faculty of Science at Zulfi, Qassim University, P.O. Box: 1712, 11932, Saudi Arabia

[2] Chemistry Department, Sohag University, Sohag, 82524, Egypt; E-Mail: omran2002@mailcity.com

References

1. Omran, O.A. The Reaction of Diethyl Bromomalonate with p-tert-butylthiacalix[4]arene: An Approach to Asymmetrical Derivatives. *Molecules* **2009**, *14*, 1755–1761.

© 2009 by the authors; licensee Molecular Diversity Preservation International, Basel, Switzerland. This article is an open-access article distributed under the terms and conditions of the Creative Commons Attribution license (http://creativecommons.org/licenses/by/3.0/).
